# Benefits, challenges and obstacles of adaptive clinical trial designs

**DOI:** 10.1186/1750-1172-6-79

**Published:** 2011-11-30

**Authors:** Shein-Chung Chow, Ralph Corey

**Affiliations:** 1 Department of Biostatistics and Bioinformatics, Duke University School of Medicine, Durham, North Carolina, USA; 2 Department of Medicine, Duke University School of Medicine, Durham, North Carolina, USA

**Keywords:** Flexibility, Efficiency, Well-understood design, Less well-understood design, Group sequential design, Adaptive dose finding, Two-stage seamless adaptive design

## Abstract

In recent years, the use of adaptive design methods in pharmaceutical/clinical research and development has become popular due to its flexibility and efficiency for identifying potential signals of clinical benefit of the test treatment under investigation. The flexibility and efficiency, however, increase the risk of operational biases with resulting decrease in the accuracy and reliability for assessing the treatment effect of the test treatment under investigation. In its recent draft guidance, the United States Food and Drug Administration (FDA) expresses regulatory concern of controlling the overall type I error rate at a pre-specified level of significance for a clinical trial utilizing adaptive design. The FDA classifies adaptive designs into categories of well-understood and less well-understood designs. For those less well-understood adaptive designs such as adaptive dose finding designs and two-stage phase I/II (or phase II/III) seamless adaptive designs, statistical methods are not well established and hence should be used with caution. In practice, misuse of adaptive design methods in clinical trials is a concern to both clinical scientists and regulatory agencies. It is suggested that the escalating momentum for the use of adaptive design methods in clinical trials be slowed in order to allow time for development of appropriate statistical methodologies.

## Introduction

In pharmaceutical/clinical research and development, clinical trials are conducted for evaluation of safety and efficacy of a test treatment under investigation. In the past several decades, it has become evident that increased spending does not reflect an increased success rate in new product development. Woodcock (2004) suggested that the low success rate of pharmaceutical development could be due to several issues. One of the most critical of these is the rapidly escalating costs of the clinical trials required for regularity approval As a result, the United States Food and Drug Administration (FDA) kicked off a *Critical Path Initiative *in early 2000s to assist sponsors in identifying the scientific challenges underlying the medical product pipeline problems [1].

In 2006, the FDA released a *Critical Path Opportunities List *that outlines 76 initial projects (six broad topic areas) to bridge the gap between the quick pace of new biomedical discoveries and the slower pace at which those discoveries are currently developed into therapies. Among the 76 initial projects, the FDA calls for advancing innovative trial designs, especially for the use of prior experience or accumulated information in trial design, which was interpreted as an encouragement for the use of adaptive design methods in clinical trials. Since then, the potential use of adaptive clinical designs in clinical research have been increasingly discussed due to its *flexibility *and *efficiency *for identifying potential signal or trend of clinical benefit of the test treatment under investigation [2,3]. In addition, it is believed that the use of adaptive trial design will not only increase the probability of success of clinical development but also shorten the time of clinical development.

In February 2010, a draft guidance on adaptive design clinical trials by the FDA was circulated for comments. This draft guidance is a document describing the potential use of adaptive designs in clinical trials. It is generally viewed as supportive of the use of adaptive designs if they are employed properly. The FDA draft guidance is not a specific guidance for the implementation of adaptive designs in clinical trials [4,5]. It, however, should be noted that adaptive designs have been used at times in confirmatory contexts, for the most part cautiously, limited to changes such as sample size re-estimation and treatment arm consolidation in the early phase of clinical development where there is more uncertainty and regulatory concerns are minimized. The FDA classifies adaptive designs into *well-understood *designs and *less well-understood *designs. Well-understood design refers to the typical group sequential design, which has been employed in clinical research for years. Less well-understood designs include the adaptive dose finding and two-stage phase I/II (or II/III) seamless adaptive designs. Many scientific issues surrounding the less well-understood designs are posted in the draft guidance without recommendations for resolution. This raises the question whether the use of adaptive design methods in clinical trials (especially for those less well-understood designs) is ready for implementation in practice.

In the next section, a definition of adaptive design by the FDA and most commonly considered adaptive designs are brief described. Sections 3 provide clinical, statistical, and regulatory perspectives on the use of adaptive design methods in clinical trials, respectively. Section 4 discusses major challenges and obstacles when implementing adaptive designs in clinical trials. A concluding remark and future perspectives regarding adaptive clinical trial designs are given in Sections 5 and 6, respectively.

## Adaptive Clinical Trial Designs

### What is adaptive design?

In clinical trials, it is not uncommon to modify trial and/or statistical procedures through protocol amendments during the conduct of clinical trials based on either external information (e.g., safety concerns raised in the medical literature) or review of interim data. The purpose is not only to efficiently identify clinical benefits of the test treatment under investigation, but also to increase the probability of success of clinical development. In this article, we will refer to the *adaptations *(or modifications) made to the trial and/or statistical procedures as the adaptive design methods. Thus, an *adaptive design *could be defined as a design that allows modifications to a trial and/or the statistical procedures of a trial after its initiation without undermining the validity or integrity of the trial [6]. In their recent publication, with the emphasis on *by design *adaptations only (rather than *ad hoc *adaptations), the Pharmaceutical Research Manufacturer Association (PhRMA) Working Group on Adaptive Design refers to an adaptive design as a clinical trial design that uses accumulating data to determine how to modify aspects of an ongoing study without undermining the validity and integrity of the trial [7]. In contrast the FDA defines an adaptive design as a study that includes a *prospectively *planned opportunity for modification of one or more specified aspects of the study design and hypotheses based on analysis of (usually interim) data from subjects in the study [4]. An adaptive design is also known as a *flexible *design by the European Medicines Agency (EMA) [8,9].

### Types of adaptive designs

*By design *adaptations may not be as flexible as they are meant to be when there is a prospectively planned adaptation embedded in the original statistical plan. In contrast, *ad hoc *adaptations via protocol amendments reflect real clinical practices, which may give clinical investigators increased flexibility for identifying possible clinical benefits of the test treatment under investigation. However, ad hoc adaptations may also increase the chance of misuse or abuse of the adaptive trial designs. Retrospective adaptations are used to develop the most appropriate statistical methods for data analysis without undermining the validity and integrity of the trial. The most commonly considered adaptive designs in clinical trials include, but are not limited to: an adaptive randomization design, a group sequential design, a flexible sample size re-estimation design (also known as an N-adjustable design), a drop-the-loser design (also known as a pick-the-winner design), an adaptive dose finding (escalation) design, a biomarker-adaptive design (also known as an enrichment target clinical trial design), an adaptive treatment-switching design, a hypothesis-adaptive design, a two-stage phase I/II (or phase II/III) seamless adaptive trial design, and a multiple adaptive design (which is a combination of any of the above adaptive designs). Detailed description of these adaptive designs and their advantages and limitations can be found in [2,3].

### Some examples

In this section, to provide a better understand of adaptive designs in clinical trials, examples concerning the use of well-understood adaptive design and less well-understood adaptive design in clinical research and development are given.

**Example 1: Group sequential design **(well-understood design) - Suppose a pharmaceutical company is interested in conducting a clinical trial utilizing a group sequential trial design with a planned interim analysis to assess safety (tolerability) and efficacy (failure rate) of a study drug in treating patients with asymptomatic adenovirus viremia. The primary objective is to test for statistical significance in the detection of a 25% difference in failure rate between the test drug and a placebo assuming that the failure rate of the placebo group is 50%. If possible the sponsor would like to stop the trial early due to efficacy/futility. Sample size calculation was performed based on the primary study endpoint of rate of failure at 12 weeks post randomization using the method of individual p-values proposed by [10]. At interim analysis, the following stopping rules based on individual p-values are considered:

Stop for efficacy if *T*_1 _≤ *α*_1_;

Stop for futility if *T*_1 _≤ *β*_1_;

Continue with adaptation if *α*_1 _<*T*_1 _≤ *β*_1_;

where *α*_1 _and *β*_1 _(*α*_1 _<*β*_1_) are the efficacy and futility boundaries at interim (stage 1), respectively, and *T*_1 _is the test statistic (based on individual p-value) to be used at interim analysis. Based on the above stopping boundaries and individual p-values, it can be shown that *α *= *α*_1_+*α*_2_(*β*_1_-*α*_1_). Thus, we choose the efficacy and futility boundaries as follows

$$\alpha_{1}=0.005,\beta_{1}=0.40,\alpha_{2}=0.0506$$

for controlling the overall type I error rate at the 5% level of significance, where α_2 _is the significance level at the end of the study. Table 1 gives sample sizes required for achieving various desired powers (e.g., 80%, 85%, and 90%) under the assumptions that (1) the failure rate of the placebo group is 60%, 55%, 50%, 45%, or 40% (2) the clinically meaningful difference is 50% of the placebo failure rate, and (3) the randomization ratio is either 1:1 or 2:1. As it can be seen from Table 1 a total of 132 subjects (88 subjects in the test group and 44 in the placebo group) are required for achieving an 80% power for detecting a 25% difference in failure rate at the 5% level of significance assuming that (1) the randomization ratio is 2:1 and (2) the true failure rate of the placebo is 50%.

**Table 1:** Sample Size Calculation

Randomization Ratio	Failure Rate		Power		
		
	Placebo	Test	80%	85%	90%
1:1	60%	30%	80 (40)	90 (45)	106 (53)
	55%	27%	90 (45)	102 (51)	120 (60)
	50%	25%	110 (55)	126 (63)	148 (74)
	45%	22%	126 (63)	144 (72)	168 (84)
	40%	20%	158 (79)	180 (90)	212 (106)
					

2:1	60%	30%	93 (62)	105 (70)	123 (82)
	55%	27%	105 (70)	120 (80)	141 (94)
	50%	25%	132 (88)	150 (100)	174 (116)
	45%	22%	150 (100)	171 (114)	201 (134)
	40%	20%	189 (126)	216 (144)	255 (170)

Note: 1. The numbers are the total sample sizes required, while the numbers in the parentheses are the sample sizes required for the test group.

2. Sample sizes do not adjust for possible dropouts.

As discussed above, group sequential design enjoys the flexibility/benefits of stopping the trial early and sample size re-estimation. However, how to control the overall type I error rate at a pre-specified level of significance when there is a possible population shift due to (i) additional adaptations at interim and/or (ii) protocol amendments has become one of the major challenges/obstacles for the well-understood design.

**Example 2: Adaptive dose escalation design **(less well-understood design) - Suppose a pharmaceutical company is planning a phase I dose escalation study to evaluate an intravenous administration of a study drug for patients with a specific type of cancer. The primary objective of this dose escalation trial is to determine the maximum tolerable dose (MTD) of the study drug. The identified MTD will be considered as the optimal dose for subsequent clinical trials conducted for later phase clinical development. The sponsor has two choices for the intended dose escalation study: an algorithm-based traditional dose escalation rule (TER) design and a model-based continual re-assessment method (CRM) design.

For the algorithm-based trial design, the traditional escalation rule is to enter three patients at a new dose level and then enter another three patients when a dose limiting toxicity (DLT) is observed. The assessment of the six patients is then performed to determine whether the trial should be stopped at the level or to escalate to the next dose level. Thus, this trial design is also known as the 3+3 TER design. The model-based CRM trial design with n patients per dose level, i.e., CRM(n), can be summarized by the following steps:

Step 1: Selecting the starting dose;

Step 2: Determining dose range and number of dose levels (usually 6-8 dose levels); In the CRM trial design, the next patient will be assigned to the dose level which is close to the estimated MTD from the updated dose-toxicity model.

Step 3: Primary assumption on the dose-toxicity model. The commonly considered dose-toxicity model in cancer research is *p*(*x*) = [1+*b *exp(-*ax*)]^-1^, where *p(x) *is the probability of toxicity with dose *x*. Under the above dose-toxicity model, the MTD can be estimated by $MTD=\frac{1}{a}\ln\left(\frac{b\theta }{1-\theta }\right)$, where *θ *is the probability of DLT (DLT rate) at MTD.

Step 4: Pre-specified dose escalation rule. For example, the CRM will employ the dose escalation rules such as the number of dose levels that are allowed to be skipped is 0 and/or the minimum number of patients treated at current dose level before escalating to the next dose level.

Step 5: Pre-specified stopping rule. For example, if the maximum number of patients at a dose level have reached 6 subjects, we claim that the MTD has been achieved.

Typical adaptations applies to the 3+3 TER trial design include (1) the flexibility of dose de-escalation and (2) the extension of the 3+3 TER to the a+b TER with and without dose de-escalation. For the CRM, a Bayesian approach is commonly considered. In clinical trials, although these two trial designs are commonly considered, little discussion regarding criteria for design selection are available in the literature.

For selecting an appropriate study design, two criteria based on a fixed sample size approach and a fixed probability of correctly identifying the MTD are commonly considered. For a fixed sample size, the optimal design can be chosen based on one of the following

(1) Number of DLTs expected;

(2) Bias and variability of the estimated MTD;

(3) Probability of observing DLT prior to MTD;

(4) Probability of correctly identifying the MTD.

In other words, we may choose the design with the highest probability of correctly identifying the MTD. If it is undesirable to have patients experience the DLT, we may choose the design with the smallest number of DLT expected. In practice, we may compromise by choosing the most appropriate design to meet our need. On the other hand, for a fixed probability of correctly identifying the MTD, the optimal design can be chosen based on one of characteristics described above. Thus, we may choose the design with the smallest probability of observing DLT prior to MTD. Similarly, we may compromise the above criteria for choosing the most appropriate design to meet our need.

For design selection of the proposed dose escalation trial, a clinical trial simulation was conducted under the assumptions that (1) the total simulation runs is 5,000, (2) the initial dose is 0.3 mCi/kg, (3) the dose range is from 0.3 mCi/kg to 2.8 mCi/kg assuming that the MTD is at 2.5 mCi/kg with a total number of dose levels of 6, (4) algorithm-based 3+3 TER with and without de-escalation are considered, (5) maximum dose de-escalation allowed is 1, (6) CRM(n) with n = 1, 2, and 3 are considered, where n is the number of subjects per dose level, (7) logistic dose-toxicity model is assumed, (8) DLT rate at MTD is assumed to be 1/3 = 33%, (9) Bayesian approach with uniform prior is considered for estimation of the parameters of the dose-toxicity model, (10) number of doses allowed to skip is 0. The results are summarized in Table 2. As a result, CRM(2) trial design is chosen for the proposed dose escalation trial for identifying the MTD.

**Table 2:** Summary of Simulation Results

Design	# Patients Expected (N)	# of DLT Expected	Mean MTD (SD)	Prob. of Selecting Correct MTD
"3+3" TER	15.96	2.8	1.26 (0.33)	0.526
"3+3" STER*	17.56	3.2	1.02 (0.30)	0.204
CRM(1)	10.60	3.4	1.51 (0.08)	0.984
CRM(2)	13.57	2.8	1.57 (0.20)	0.884
CRM(3)	16.37	2.7	1.63 (0.26)	0.784

* Allows dose de-escalation

CRM(n) = CRM with n patients per dose level; Uniform prior was used.

Note that the CRM(n) with n = 2 will provide a more accurate and reliable assessment of the dose-toxicity model as compared to that of the CRM(n) with n = 1. Although the CRM(n) (in conjunction with Bayesian approach) provides a more accurate and reliable approach for identifying the MTD, the validation of the dose-toxicity mode, the selection of appropriate prior, possible dose jump and overdose have become major challenges/obstacles for the investigators.

**Example 3: Two-stage phase II/III adaptive design **(less well-understood design) - A pharmaceutical company is interested in conducting a clinical trial utilizing a two-stage seamless adaptive design for evaluation of safety (tolerability) and efficacy of a test treatment for patients with hepatitis C virus (HCV) infection. The study will consist of two stages at which the first stage is for dose selection and the second stage is for establishment of non-inferiority of the selected dose from the first stage as compared to the standard of care therapy (control). The primary objectives of the study then contain study objectives at both stages. For the first stage, the primary objective is to select the optimal dose as compared to the standard of care therapy, while the primary objective of the second stage is to establish non-inferiority of the selected dose as compared to the standard of care therapy. The treatment duration is 48 weeks of treatment followed by a 24 weeks follow-up. The primary study endpoint is the sustained virologic response (SVR) at week 72, which is defined as an undetectable HCV RNA level (< 10 IU/mL) at week 72.

The proposed two-stage seamless adaptive design is briefly outline below: Stage 1: This stage is a five-arm randomized evaluation of four active dose levels of the test treatment. Qualified subjects will be randomly assigned to one of the five treatment groups at a 1:1:1:1:1 ratio. After all Stage 1 subjects have completed Week 12 of the study, an interim analysis was performed. Based upon the safety results of this analysis as well as virologic response at Weeks 12 and 24, Stage 1 subjects who have not yet completed the study protocol will continue with their assigned therapies for the remainder of the planned 48 weeks, with final follow-up at Week 72. An optimal dose will be selected based on the interim analysis results of the 12 week early virologic response (EVR), which is defined as 2-log10 reduction in HCV RNA level at Week 12, assuming that the 12 week EVR is predictive of 72 week SVR. The 12 week EVR is considered as a surrogate endpoint for the primary endpoint of 72 week SVR. Under this assumption, an optimal dose will be selected using precision analysis under some pre-specified selection criteria. In other words, the dose group with highest confidence level for achieving statistical significance (i.e., the observed difference is not by chance alone) will be selected. The selected dose will then proceed to testing for non-inferiority compared to standard of care in Stage 2. Stage 2: This stage will be a non-inferiority comparison of the selected dose from Stage 1. A separate cohort of subjects will be randomized to receive either the selected dose from Stage 1 or the standard of care treatment as given in Stage 1 in a 1:1 ratio. A second interim analysis will be performed when all Stage 2 subjects have completed Week 12 and 50% of the subjects (Stage 1 and Stage 2 combined) have completed 48 weeks treatment and follow-up of 24 weeks. Depending on the results of this analysis, including the virologic response at Weeks 12 and 24, sample size re-estimation will be performed to whether additional subjects are needed in order for achieving the desired power for establishment of non-inferiority for the selected dose.

In both stages, subjects who do not meet the study criteria for virologic response at Weeks 12 and 24, and those who do meet these criteria but then relapse at any later time through study Week 72, will discontinue study treatment and will be offered treatment, off protocol, with standard of care. For the two planned interim analyses, the incidence of EVR as well as safety data, will be reviewed by an independent data safety monitoring board (DSMB). The commonly used O'Brien-Fleming boundaries will be applied for controlling the overall Type I error rate at 5% [11,12]. Adaptations such as stopping the trial early, discontinuing selected treatment arms, and re-estimating the sample size may be applied as recommended by the DSMB. Stopping rules for the study will be designated by the DSMB, based on their ongoing analyses of the data and as per their charter.

In clinical research and development, the use of two-stage phase I/II or phase II/III adaptive seamless design has become very popular due to its flexibility and efficiency for achieving the study objectives of the intended trials. However, the development of appropriate and valid statistical methods and the control of the overall type I error under the complexity of the less well-understood design are major challenges/obstacles to clinical scientists.

### 2.4 Benefits of adaptive designs

Possible benefits for the use of adaptive design methods in clinical trials include that (1) it allows the investigator to correct wrong assumptions made at the beginning of the trial, (2) it helps to select the most promising option early, (3) it makes use of emerging external information to the trial, (4) it provides the investigator the opportunity to react earlier to surprise (either positive or negative), and (5) it may shorten the development time and consequently speed up development process. In summary, the use of adaptive design methods in clinical research and development provides the investigator the second chance to modify or re-design the trial after seeing data from the trial itself at interim or externally as recommended by the independent data monitoring committee (IDMC) of the study.

While enjoying the flexibility and possible benefits of adaptive design methods in clinical trials, it should be noted that more flexibility could lead to a less well-understood design as described in the FDA draft guidance. A less well-understood adaptive design is often more flexible and yet more complicated. Under a complicated and less well-understood adaptive design, statistical inference is often difficult, if not impossible, to obtain although valid statistical inferences for some less well-understood designs are available in the literature. As an example, Table 3 provides a summary of flexibilities and possible benefits of some less well-understood adaptive designs such as an adaptive randomization design, an adaptive dose finding design, and a two-stage phase I/II (or phase II/III) seamless adaptive design that are commonly considered in pharmaceutical/clinical research and development.

**Table 3:** Summary of Flexibility/Benefits and Challenges/Obstacles of Various Less Well-Understood Adaptive Designs

Design	Flexibility/Benefits	Challenges/Obstacles
Adaptive Randomization Design	■ Unequal probability of treatment assignment ■ Assign subjects to more promising treatment arm	■ Randomization schedule not available prior to the conduct of the trial ■ Not feasible for large trials or trials with long treatment duration ■ Statistical inference is often different, if not impossible, to obtain
		
Adaptive Dose Finding Design*	■ Drop inferior dose group early ■ Modify/add additional dose groups ■ Increase the probability of correctly identifying the MTD with limited number of subjects	■ Selection of initial dose ■ Selection of dose range under study ■ Selection criteria and decision rule ■ Risk of dropping promising dose groups
		
Two-stage Seamless Adaptive Design (either phase I/II or phase II/III)	■ Combine two studies into a single study ■ Fully utilize data collected from both stages ■ Reduce lead time between studies ■ Shorten the development time ■ Additional adaptations such as drop-the-loser, adaptive randomization, and adaptive hypotheses may be applied at the end of the 1^st ^stage	■ The control of the overall type I error rate ■ Sample size calculation/allocation ■ How to perform analysis based on combined data collected from both stages? ■ Is the O'Brien-Fleming type of boundaries feasible?

*For example, adaptive dose escalation designs for cancer trials.

## Clinical/Statistical and Regulatory Perspectives

### Clinical Operation Perspectives

From clinical operation perspectives, the use of adaptive design methods in clinical trials does reflect real clinical practices in clinical research and development. In clinical practice, the prospective (or by design) adaptations of unequal ratio of randomization, data safety monitoring, interim analysis for efficacy, stopping the trial early due to safety and efficacy/futility, and sample size re-estimation are commonly considered at the stage of protocol development. During the conduct of the trial, it is not uncommon that some concurrent (ad hoc) adaptations such as modification of inclusion/exclusion criteria, dose regimen/duration, and primary study endpoints and/or hypotheses are implemented through protocol amendments due to slow enrollment, safety concern, and the issue of lack of efficacy, respectively. Note that frequent ad hoc adaptations without consideration of the statistical implications have provided an argument for using prospectively planned adaptive design methods in clinical trials. At the end of the study, some retrospective adaptations on statistical analysis plan of the data collected from the trial are often applied prior to database lock or data unblinding.

As indicated earlier, the use of adaptive clinical trial design is very attractive due to its flexibility and efficiency for identifying optimal clinical benefits of a test treatment under investigation especially when only limited resources and/or time are available. However, before an adaptive design can be implemented, the practical issues of feasibility, validity and robustness, which have impact on the data quality and the integrity of the trial, are necessarily addressed from clinical perspectives. These practical issues are briefly described below.

For feasibility, the following questions arise and need to be addressed before the interested adaptive design can be implemented [13]: (1) Do the possible benefits outweigh the extra efforts required for implementation of the adaptive design? (2) Does the level of difficulty and the associated cost justify the gain from implementing the adaptive design? (3) Does the implementation of adaptive design delay patient recruitment and prolong study duration? (4) How often are the unblinded analyses practical and to whom should the data be unblinded? (5) How should the impact of the data monitoring committee's (DMC) decision regarding the trial (e.g., recommending an early stopping or other adaptations due to safety concern) be considered at the design stage?

For the issue of validity, it is reasonable to ask the following questions: (1) Does the unblinding cause potential bias in treatment assessment? (2) Does the implementation of an adaptive design destroy the randomness? For example, response-adaptive randomization is used to assign more patients to the superior treatment groups by changing the randomization schedule. However, for ethical reasons, the patients should be informed that the later they come into the study, the greater the chance of being assigned to the superior groups. For this reason, patients may prefer to wait for late entry into the study. This could cause bias because sicker patients might enroll earlier just because they cannot wait. When this happens, the treatment effect is confounded by the patient's disease background. A similar bias could occur for a drop-losers design and other adaptive designs.

Regarding the issue of robustness, virtually without exception, a trial cannot be conducted exactly as specified in the protocol. In practice, it is helpful to assess the issue of robustness by addressing the following questions. First, would protocol deviations and/or violations invalidate the adaptive trial design employed? For example, if an actual interim analysis was performed at a different (information) time that the scheduled one, how does it impact the type-I error of the adaptive design? How does an unexpected DMC action affect the power and validity of the design? Would a protocol amendment such as endpoint change or inclusion/exclusion change invalidate the design and analysis? Would delayed responses diminish the advantage of implementing an adaptive design such as continued re-assessment method (CRM) in an adaptive dose-escalation design and trials with a survival endpoint? In addition, what level of modifications to the trial would be acceptable to the regulatory authorities? Does the adaptive design have adequate theoretical support? Does implementation of the adaptive design deviate from the theoretical model? Is the adaptive design robust against major protocol deviations? Does the data unblinding actually cause bias in assessment?

### Statistical Perspectives

From a statistical point of view, major adaptations or modifications to trial and/or statistical procedures could (1) introduce operational bias/variation to data collection, (2) result in a shift in the target patient population in terms of either location or scale parameter, and (3) lead to inconsistency between hypotheses to be tested and the corresponding statistical tests.

In clinical trials, any modifications made to the trial and/or statistical procedures may introduce operational bias and/or variation to the data collection. The sources of bias/variation can be classified into four categories, namely (1) expected and controllable such as changes in laboratory testing procedures and/or diagnostic procedures, (2) expected but not controllable such as change in study dose and/or treatment duration, (3) unexpected but controllable such as patient non-compliance, and (4) unexpected and uncontrollable which is the random error in observing the clinical responses/outcomes [14]. For good clinical practice, we should make every attempt not only to identify but also to minimize/control possible operational bias/variation whenever possible.

As indicated in [2], significant or major modifications made to the trial could result in a shift in target patient population (i.e., from the target patient population to a similar but slightly different target patient population). It is then a concern whether (significant or major) changes made to the trial have led to a totally different trial with a similar but different target patient population. Consequently, we may not be able to answer the medical/scientific questions that the original trial intended to address. Thus, it is of interest to determine whether statistical inference obtained based on clinical data collected from the actual patient population could be applied to the originally planned target patient population.

In addition, the misuse and/or abuse of the adaptive design methods in clinical trials could lead to inconsistencies between hypotheses to be tested and the corresponding statistical tests where (1) there are wrong tests for the right hypotheses (the validity is a concern), (2) there are right tests for the wrong hypotheses (an evidence of the misuse of certain adaptations), (3) there are wrong tests for the wrong hypotheses (an evidence of abuse of the adaptive design methods), and (4) there are right tests for the right hypotheses with insufficient power [14].

In clinical investigation, a pre-study power analysis for sample size calculation is always performed for achieving a desired power for correctly detecting a clinically meaningful difference (or treatment effect) at a pre-specified level of significance. The ultimate goal is to make sure that the observed difference has both clinical and statistical meaning in the sense that (1) it is of clinical importance, (2) it is not by chance alone, and (3) it is reproducible. The pre-study power analysis can only be done under a valid statistic derived under the null hypothesis. Thus, for a given adaptive design, valid statistical methods are necessary to ensure the success of the clinical trials utilizing adaptive trial designs, especially for those less well-understood adaptive designs.

### Regulatory Perspectives

As indicated earlier, the use of adaptive design methods based on accrued data in clinical trials may introduce operational bias, which is a great concern to the regulatory agencies in the review/approval process of the regulatory submission. One of the major concerns is that the use of adaptive trial designs (especially for those less well-understood designs) may not be able to preserve the overall type I error rate at the pre-specified level of significance. In addition, p-values may not be correct and the corresponding confidence intervals for the treatment effect may not be reliable. Moreover, adaptations may result in a totally different trial that is unable to address the medical questions that the original study intended to answer.

In clinical trials, it is recognized that the use of adaptive design methods (either by design adaptation or ad hoc adaptation) may introduce operational biases such as selection bias, method of evaluations, early withdrawal, and modification of treatments. Consequently, the adaptation employed may inflate type I error rate [15]. In practice, operational biases could be translated to information (assessment) biases, which may include patient enrollment, differential dropouts in favor of one treatment, crossover to the other treatment, protocol deviation due to additional medications or treatments, and differential assessment of the treatments [16]. Commonly seen adaptations which have an impact on the type I error rate include, but are not limited to, sample size adjustment at interim, sample size allocation to treatments, delete/add or change treatment arms, shift in target patient population such as changes in inclusion/exclusion criteria, change in statistical test strategy, change in study endpoints, and change in study objectives such as the switch from a superiority trial to a non-inferiority trial [15]. As a result, it is difficult to interpret the clinically meaningful effect size for the treatments under study [16].

These regulatory concerns have led to the development of valid statistical methods under various less well-understood adaptive designs. As a result, the escalating momentum behind adaptive clinical trial designs continues moving forward since the FDA draft guidance was distributed for comments in February, 2010.

## Major Challenges and Obstacles

Despite the attractive characteristics of flexibility and efficiency of adaptive design trial designs in clinical trials, some concerns regarding the quality, validity and integrity of the trials arise, which have resulted in major challenges and obstacles to the investigators, clinical scientists and biostatisticians when implementing adaptive design methods in clinical trials. In this section, some challenges and obstacles, clinical trial simulation, and software application packages are discussed (see also Table 3).

### Well-understood design

In clinical trials, a group sequential design is often considered for (1) early stopping for clinical benefit or harm, (2) early stopping for futility, (3) sample size re-estimation, and (4) re-designing the study in mid-stream [17]. As indicated in the FDA draft guidance, group sequential designs are considered well-understood when design characteristics 1 or 2 are applied but not when design characteristics 3 or 4 are incorporated. The well-understood group sequential design is very popular due to the following two reasons. First, clinical endpoint is a moving target. The sponsors and/or investigators may change their mind regarding clinically meaningful effect size after the trial starts. Second, it is a common practice to request a small budget at the design and then seek for supplemental funding for increasing the sample size after seeing the interim data. To protect the overall type I error rate in an adaptive design with respect to changes in some design parameters, many authors have proposed procedures using observed treatment effects. This leads to the justification for the commonly used two-stage adaptive design, in which the data from both stages are independent and the first data are used for adaptation. When there is a shift in the location and/or scale parameters of the target patient population due to major changes in protocol amendments (e.g., major changes in eligibility criteria), however, standard methods for the well-understood group sequential design may not be valid. In this case, "*How to protect the overall type I error rate with respect to changes in some design parameters?" *has become a challenge to biostatisticians.

### Less well-understood designs

In practice, two-stage phase II/III seamless adaptive designs with different study objectives and/or different study endpoints at different stages are considered less well-understood designs in the sense that (1) valid statistical methods are yet to be developed, and (2) the impact of additional adaptations on statistical inference is unknown [18,19]. Under the two-stage adaptive design, "*How to perform sample size calculation/allocation at the planning stage?", "How to control the overall type I error rate at a pre-specified level of significance?"*, and "*How to combine data collected from both stages for a final data analysis*?" are major challenges to biostatisticians. In addition, it is a concern when there is a population shift due to protocol amendments, which will make the less well-understood design even more complicated and lesser well-understood.

### Clinical Trial Simulation

Clinical trial simulation is a process that uses computers to mimic the conduct of a clinical trial by creating virtual patients to extrapolate (or predict) clinical outcomes for each virtual patient based on the pre-specified models [20,21]. The primary objective of clinical trial simulation is multi-fold. First, it is used to monitor the conduct of the trial, project outcomes, anticipate problems and recommend remedies before it is too late. Second, it is used to extrapolate (or predict) the clinical outcomes beyond the scope of previous studies from which the existing models were derived using the model techniques. Third, it is used to study the validity and robustness of the trial under various assumptions of study designs. Clinical trial simulation is often conducted to verify (or confirm) the models depicting the relationships between the inputs such as dose, dosing time, patient characteristics, and disease severity and the clinical outcomes such as changes in the signs and symptoms or adverse events within the study domain. In practice, clinical trial simulation is often considered a way of predicting potential clinical outcomes under different assumptions and various design scenarios at the planning stage of a clinical trial for a better planning of the actual trial. However, clinical trial simulation is useful only when based on a well-established predictive model under certain assumptions [21]. "*How to validate the assumed predictive model for clinical trial simulation?" *is a major challenge to both investigators and biostatisticians.

### Software Packages

As indicated earlier, more adaptations give the investigator more flexibility in identifying best clinical benefits of the test treatment under investigation. However, a multiple adaptive design with more adaptations could be very complicated and consequently appropriate statistical methods for assessment of the treatment effect may not be available and are difficult, if not impossible, to obtain. Thus, one of the major obstacles for implementing adaptive design methods in clinical trials is that appropriate statistical methods are not well established with respect to various adaptations. Current software packages such as SAS cannot be applied directly and hence are not helpful. Although there are some software available in the marketplace such as ExpDesign Studio [22], EastSurvAdapt [23], and ADDPLAN (http://www.addplan.com), which cover certain types of adaptive trial designs, new software packages for adaptive design methods in clinical trials are necessary to assist in implementing adaptive trial designs in clinical trials [24]. An overview of software available for group sequential and adaptive designs can be found in [25].

## Concluding Remarks

In clinical trials, although the flexibility of modifying study parameters is very attractive to clinical scientists, several scientific (clinical, statistical, and regulatory) questions/concerns arise. First, what level of modifications to the trial procedures and/or statistical procedures would be acceptable to the regulatory authorities? Second, what are the regulatory requirements and standards for the review and approval process of clinical data obtained from adaptive clinical trials with different levels of modifications to trial procedures and/or statistical procedures of on-going clinical trials? Third, has the clinical trial become a totally different trial after the modification of the trial procedures and/or statistical procedures for addressing the study objectives of the originally planned clinical trial? These concerns should be addressed by the regulatory authorities before the adaptive design methods can be widely accepted in clinical research and development. As a result, guidelines for *specific *adaptive design methods must be developed in order to avoid every intentional or unintentional manipulation of the adaptive design results in clinical trials. The guidelines should describe in detail not only the standards for use of specific adaptive design methods in clinical trials, but also the level of modification in an adaptive design that is acceptable to the regulatory agencies. In addition, any changes in the process of regulatory review/approval should be clearly indicated in such guidelines. It should be noted that the adaptive design methods have been used in the review/approval process of regulatory submissions for years, though it may not have been recognized until recently. As indicated earlier, most adaptive clinical trials designs for clinical investigation of a test treatment under investigation that are of particular interest to the investigators are considered less well-understood designs. For some (complicated) less well-understood designs, statistical methods are yet to be developed. The use of an independent data safety monitoring board (DSMB) will not only help to prevent the investigator from misuse and/or abuse of the adaptive design methods, but also to ensure the quality, validity, and integrity of the trials utilizing adaptive designs [26].

## Future Perspectives

We are moving in the right direction and yet there is still a long way to go until we are able to address all of the scientific issues from clinical, statistical, and regulatory perspectives as described earlier. Detailed design-specific guidances (e.g., guidances regarding sample size calculation/allocation and statistical/clinical considerations for a two-stage phase I/II or phase II/III seamless adaptive trial design) must be developed by the regulatory agencies before implementation of adaptive design methods in pharmaceutical/clinical research and development. In addition, qualification, composition, role/responsibility, and function/activity of an independent data monitoring committee for implantation of adaptive trial design need to be established for an objective and unbiased assessment of the treatment effect of the drug under investigation. Thus, from future perspectives, it is suggested that the escalating momentum for the use of adaptive design methods in clinical trials proceed with caution. At the same time, valid statistical methods for interested adaptive designs with various adaptations should be developed to prevent the possible misuse and/or abuse of the adaptive design methods in clinical trials.

## Competing interests

The authors declare that they have no competing interests.

## Authors' contributions

Both SC and RC participated in the development of the early version of the manuscript. SC carried out the examples. RC provided medical input and constructive discussion. All authors read and approved the final manuscript.
